# Unique Presentation of Hematuria in a Patient with Arterioureteral Fistula

**DOI:** 10.1155/2016/8682040

**Published:** 2016-05-12

**Authors:** Tomas Mujo, Erin Priddy, John J. Harris, Eric Poulos, Mahmoud Samman

**Affiliations:** ^1^Department of Radiology, University of Louisville Hospital, Louisville, KY 40202, USA; ^2^University of Louisville School of Medicine, Louisville, KY 40202, USA

## Abstract

Active extravasation via an arterioureteral fistula (AUF) is a rare and life-threatening emergency that requires efficient algorithms to save a patient's life. Unfortunately, physicians may not be aware of its presence until the patient is in extremis. An AUF typically develops in a patient with multiple pelvic and aortoiliac vascular surgeries, prior radiation therapy for pelvic tumors, and chronic indwelling ureteral stents. We present a patient with a left internal iliac arterial-ureteral fistula and describe the evolution of management and treatment algorithms based on review of the literature.

## 1. Introduction

Arterioureteral fistula is a direct communication between an artery and ureter that presents with hematuria. A fistula may form between the aorta and common iliac, external iliac, and internal iliac arteries. AUF can be seen in the setting of prior oncologic pelvic surgery, pelvic exenteration, vascular surgeries of the pelvis, and pelvic irradiation therapy. Cases can also be seen following urologic diversion procedures and in patients with indwelling ureteral stents requiring frequent stent exchanges.

## 2. Case Report

We present the case of a 54-year-old woman with past medical history of stage IIB squamous cell carcinoma of the cervix, pelvic radiation therapy 16 years earlier, chronic renal insufficiency, chronic pyelonephritis, solitary left kidney, left ureteral stricture, frequent ureteral stent exchanges, left percutaneous nephrostomy tube, and colovesical and vesicovaginal fistulae. Her past surgical history includes total abdominal hysterectomy and bilateral salpingo-oophorectomy, sigmoid colostomy with Hartmann's pouch, ileostomy, and right nephrectomy.

She developed bright red blood from the left ureteral orifice after removal of a metallic ureteral stent during an exchange procedure at an outside hospital. Subsequently, she had a retrograde pyelogram, which showed no vascular blush or active extravasation of blood. The only positive finding at that time was a large blood clot within the left renal pelvis. Vascular surgery evaluated the patient and was unable to provide the necessary vascular interventions. A technetium pertechnetate tagged red blood cell scan revealed no gastrointestinal source for bleeding. Four days later, the patient began passing bright red blood and clots from her left nephrostomy tube. Her hemoglobin dropped two grams/dL, requiring transfusion of 2 units of packed red blood cells.

Later that day the patient was transferred to our hospital for further management. Active hematuria via the Foley catheter and left nephrostomy tube had resolved at the time of admission to our facility. Her vital signs were within normal limits. Review of the computed tomography (CT) of the abdomen and pelvis without intravenous contrast from the outside institution stated that there was extensive chronic soft tissue thickening at the site of previous surgical operations, likely an area of fistula formation. The patient was placed on continuous bladder irrigation.

**Figure 1:**
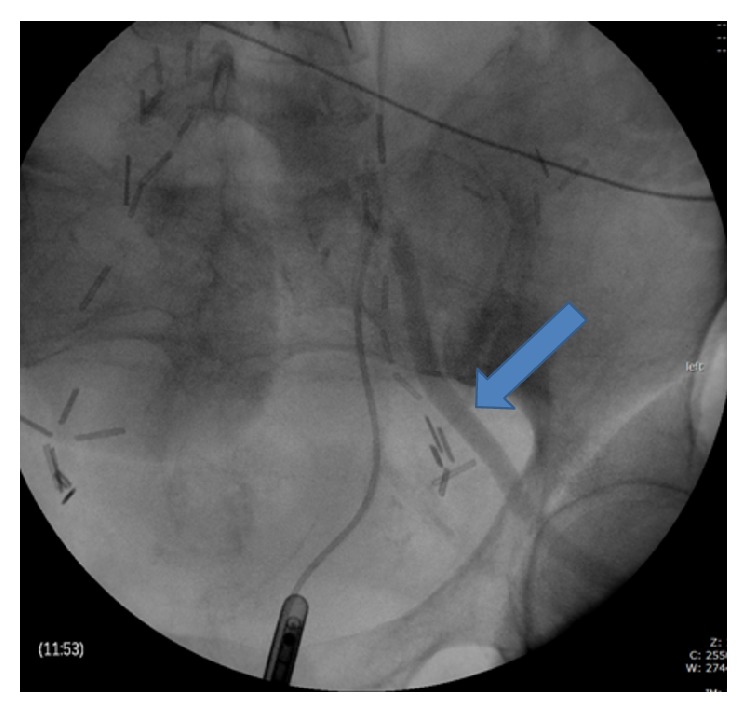
Left ureteral retrograde pyelogram demonstrates contrast opacification of the left external iliac artery (solid blue arrow) consistent with left ureteral-arterial fistula. Scattered surgical clips are present throughout the pelvis.

Three days later her hemoglobin was stable, and cystoscopy and left retrograde pyelogram were performed. At cystoscopy, a large blood clot was irrigated out and removed. After removal of the clot, a large vesicovaginal fistula was identified in the posterior aspect of the bladder. No other lesions were visualized. Subsequently, the left ureteral orifice was intubated with a catheter. Contrast was injected into the ureter and a vascular blush and contrast opacification of the left external iliac artery were identified (Figure 1). At this point, the procedure was aborted and a 3-way Foley catheter was placed for continuous bladder irrigation.

Interventional Radiology was consulted emergently to evaluate and treat the patient. The patient was allergic to contrast media and was premedicated emergently with Benadryl® (West Ward Pharmaceuticals Corp.) and Solu-Cortef® (Pharmacia and Upjohn Company). Visipaque® (GE Healthcare Inc.) contrast was used for the procedure. Initially the left groin was accessed and a hand injection performed via a five-French measuring pigtail catheter in the left proximal common iliac artery. The left proximal external iliac artery demonstrated severe stenosis. At this point the right groin was accessed and a six-French vascular sheath was placed over a 0.035-inch wire, and an arteriogram of the pelvic arteries from the right common femoral artery approach was performed. This arteriogram was helpful to estimate the length and the severity of the stenosis of the left iliac arteries. It also aided to minimize traumatic injury to the left iliac arteries during their recanalization.

**Figure 2:**
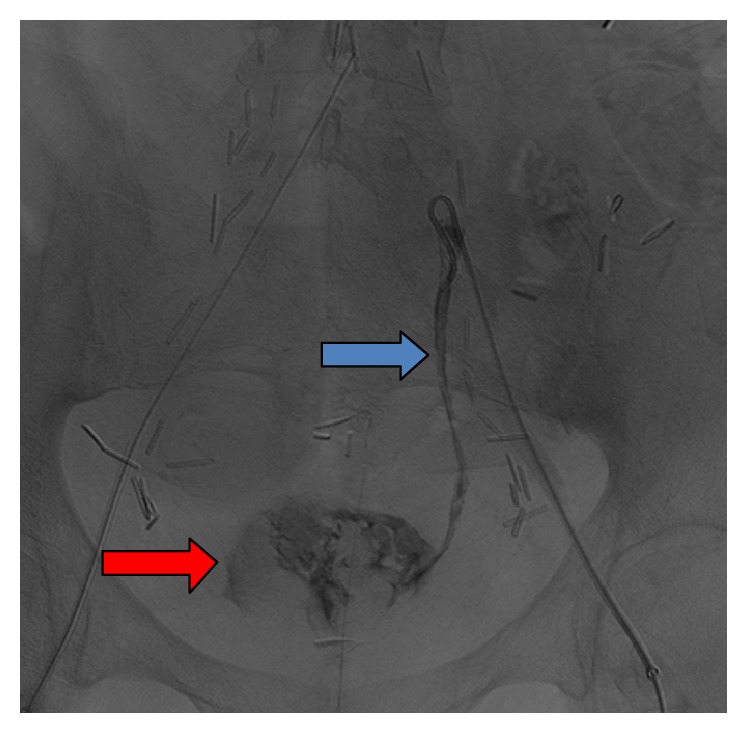
Attempted left internal iliac artery selective arteriogram with a 5 F SOS catheter shows the catheter tip in the left ureter and brisk flow of contrast into the left ureter (solid blue arrow) flowing caudad into the urinary bladder (solid red arrow). This confirms the presence of an internal iliac arterioureteral fistula.

Then, after successful recanalization of the left iliac arteries, a pigtail catheter was inserted over the wire into the distal abdominal aorta and a pelvic arteriogram was performed. Subsequently, the left pigtail catheter was exchanged for a 5 Fr SOS selective catheter and attention was directed to the left internal iliac artery. An attempt was made to select the left internal iliac artery, and then contrast was injected which demonstrated the catheter to be in the left ureter instead of the left internal iliac and the arteriogram demonstrated brisk flow of contrast into the left ureter, confirming the presence of an internal iliac arterioureteral fistula (Figure 2). The arterial catheter ending up into the ureter is presumably dangerous and can cause a sudden enlargement of the fistula.

**Figure 3:**
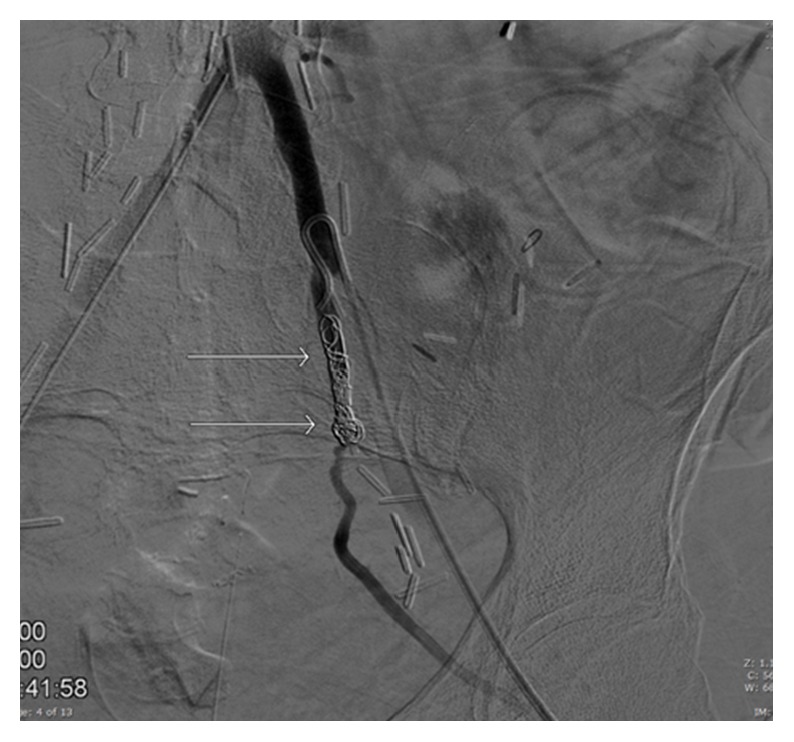
Four platinum coils were used to embolize the left proximal internal iliac artery (white arrows).

**Figure 4:**
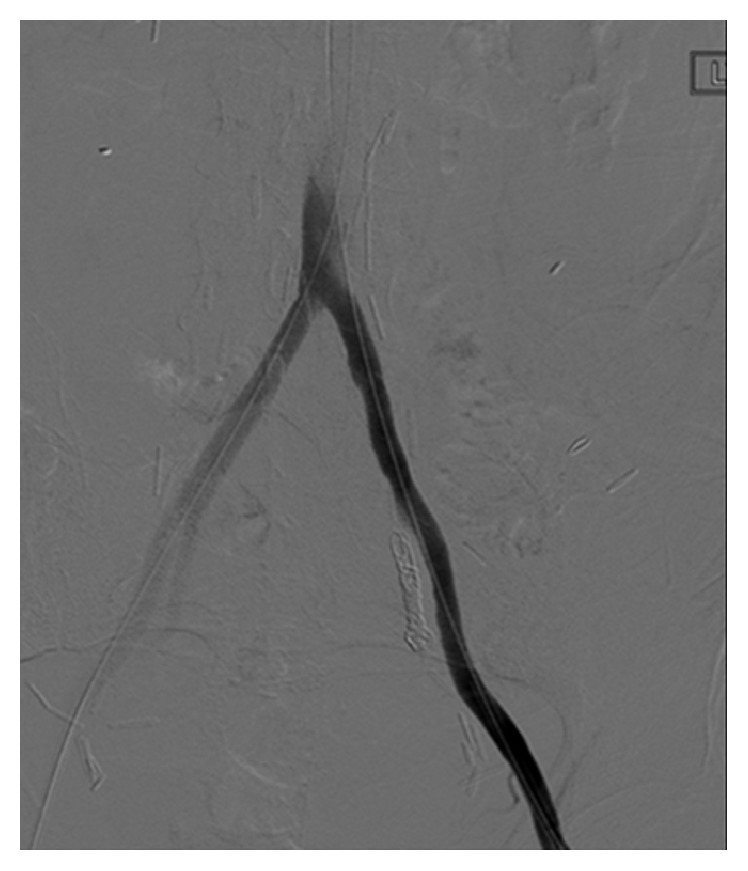
Left iliac arteriogram after left internal iliac embolization and placement of an 8 mm × 5 cm Gore® Viabahn® stent from the mid left common iliac artery to the proximal external iliac artery shows no opacification of the left internal iliac artery and elimination of the left internal iliac arterioureteral fistula.

The SOS catheter was advanced to the common iliac artery and another attempt was made utilizing the road map technique to select the left internal iliac artery for the purpose of embolization planning. Essentially, embolization of the left internal iliac artery was planned to prevent formation of a type II endoleak after placement of a covered stent from the common to external iliac arteries. The left internal iliac artery was successfully selected. Subsequently, four platinum coils were used to embolize the left internal iliac artery (Figure 3) and the postembolization arteriogram demonstrated no flow into the left internal iliac artery or left ureter (Figure 4).

Attention was then directed to the severe stenosis of the left proximal external iliac artery. A 7 mm × 4 cm Dorado® (BARD Peripheral Vascular) angioplasty balloon was utilized to dilate the stenosis followed by placement of an 8 mm × 5 cm Gore Viabahn Endoprosthesis which extended from the mid left common iliac artery to the proximal external iliac artery (Figure 4). The patient was discharged home in stable condition three days after the procedure. No follow-up documentation or imaging is available in our electronic medical system.

## 3. Discussion

The arterioureteral fistula is a rare and often life-threatening complication that requires a high index of suspicion for early detection and to discern it from other causes of hematuria. Virtually all patients present with gross hematuria, the timing of which is variable: some develop episodic hematuria requiring intermittent transfusion, others demonstrate no clinical signs prior to massive hematuria with hemodynamic instability, and others only present with hematuria at the time of stent exchange [1]. Increasing numbers of case reports have emerged over the recent years describing the expansion of endovascular interventions and the rare endoureteral intervention as treatment algorithms have evolved [2, 3].

There is a prominent identifiable subset of patients most commonly affected. Of the 118 patients studied in multi-institutional analysis, 73.7% had a history of chronic indwelling ureteral stents, 70.3% had a history of malignancy, and 69.5% had undergone prior pelvic surgery [4]. Women were more commonly affected than males (approximately 3 : 2 ratio), which is logical considering many of these patients have had prior pelvic surgery or radiation [4]. A smaller single-institution study [2] added prior radiation therapy to the predisposing factors. Of the patients identified with AUF, 75% had undergone prior radiation treatment.

The increase in incidence of AUF is almost exclusively related to the increased number of secondary fistulae. Primary fistulae are overwhelmingly related to inflammation from an adjacent aneurysm or pseudoaneurysm. In one review series, of the ten primary fistulae identified, nine were related to aortoiliac aneurysm, while the additional patient had an arteriovenous malformation [5]. Rare instances of primary fistulae during pregnancy have been identified postmortem secondary to ureteropelvic obstruction from advanced pyelonephritis. This etiology has disappeared, likely because of aggressive antibiotic use in the setting of urinary tract infection during pregnancy [5].

While the pathophysiology of fistula formation is still not fully understood, various contributing factors are thought to play a role. External beam radiation and pelvic surgery may disrupt the vasa vasorum, making larger vessels more prone to necrosis and subsequent fistula formation [1, 6]. A similar phenomenon is believed to occur in ureters, causing ureteral devascularization in patients subjected to the same interventions [7, 8]. A mechanical component also is thought to contribute: arterial pulsations cause chronic microtrauma to the overlying ureter, particularly those having been chronically stented. This mechanical microtrauma may then progress to pressure necrosis and arterial-ureteral communication [7]. Krambeck et al. state that ureteral stent size plays a role in fistula formation. For instance, the smaller diameter 7 F ureteral stent provides the same flow rate as a 12 F stent, with potential reduced risk of ureteral dilatation, wall compression, and ischemic changes.

Even with high clinical suspicion, diagnostic confirmation is challenging for a variety of reasons, including the same basis of episodic symptoms: intermittent thrombus occlusion of the fistulous tract [9]. According to the most recent review of the literature, selective arteriography is the most effective modality for diagnosis [4, 10]. An older study cites arteriography sensitivity of 50% [11]. Interestingly, in our case, we did not see the arterial-ureteral communication during our initial pelvic arteriogram. Rather, we saw the arterial-ureteral communication only when we tried to select the left internal iliac artery and unintentionally selected the left ureter. Thus, even when not visualized with contrast, an arterioureteral fistula may be present. The friable, fragile nature of the tissues separating the left ureter from the left iliac arteries likely explains the inconsistent visualization of the AUF and the intermittent nature of the patient's hematuria.

Several studies [2, 4, 5, 12–14] suggest that provocative angiography, by way of stent removal or manipulation of urinary catheter, improves sensitivity, although with obvious needed preparation for potential massive hemorrhage. Computed tomography is not typically revealing, although Krambeck et al. stated it was diagnostic in half of the patients who underwent the aforementioned imaging modality in their study cohort [1]. Bleeding from a ureteral orifice during cystoscopy is a nonspecific finding, but should heighten suspicion in the appropriate clinical scenario. Suggestion has been made for anterograde or retrograde ureterogram to demonstrate contrast extravasation from the ureter into the arterial circulation [4]; however, the pressure gradient makes this visualization less likely.

Open surgical repair, via local reconstruction, ligation with or without extra-anatomic reconstruction, or ligation of the internal iliac artery was commonly used prior to the advent of endovascular techniques [5]. Most often, femoral crossover bypass was utilized given the potential infection risk [5]. Common complications from open surgical management include enterocutaneous fistulae or wound infection [1]. In addition to the morbidity, which can often accompany open surgical repair, these complex patients are often not ideal operative candidates, due to a mummified pelvis from prior surgeries and radiation, thus allowing for advantageous utilization of endovascular techniques.

With the first use of iliac artery stent grafts in 2004, many patients have been treated definitively with endovascular stenting. Despite this utilization of iliac artery stents for over 10 years, standardized antibiotic prophylaxis has yet to be established. Other endovascular treatment options include isolated coil embolization of the iliac artery or most commonly exclusion of the fistula with a covered stent graft [12]. A few studies describe successful treatment with isolated coil embolization and self-expanding polytetrafluoroethylene covered stent graft via the ureter by Inoue et al. and Horikawa et al., respectively [3, 15]; however, the efficacy of this method has not been demonstrated in other instances.

A single center retrospective review of arterioureteral fistulas by Fox et al. did not demonstrate a clear morbidity or mortality advantage for open or endovascular management [6]. In the majority of patients, endovascular intervention is preferred given patient comorbidities, hostile surgical abdomen given multiple prior surgeries and radiation therapies, and complication rate. Prior to 1980 the mortality rate associated with AUF was estimated at 69% [1], and a mortality rate of 100% without treatment. The most recent review of the literature gives an acute mortality rate of 10–38% [4]. True long-term follow-up is still yet to be determined as the longest duration of median follow-up in studies to date is 26 months [2].

Hematuria is a nonspecific finding which should raise suspicion for arterioureteral fistula in patients with history of prior pelvic vascular or oncologic surgery, radiation therapy, and multiple ureteral stents. Diagnostic confirmation can be elusive as seen in our case, but lack of imaging confirmation should not deter diagnosis and treatment of AUF in cases of high clinical suspicion. Increased utilization of endovascular treatment in the past decade has proven useful given the complexity of the majority of patients who present with AUF.

In conclusion, continued attention to long-term morbidity and mortality is needed, particularly in reference to hematuria or fistula recurrence. Mortality due to AUF has markedly decreased over the past 30 years and may continue to decrease with increased clinical awareness and minimally invasive treatment options, which will be seen with ongoing long-term follow-up.

## Abbreviations

AUF: Arterioureteral fistula
CT: Computed tomography
IRB: Institutional review board.
